# Antiretroviral Therapy, Especially Efavirenz, Is Associated with Low Bone Mineral Density in HIV-Infected South Africans

**DOI:** 10.1371/journal.pone.0144286

**Published:** 2015-12-03

**Authors:** Joel A. Dave, Karen Cohen, Lisa K. Micklesfield, Gary Maartens, Naomi S. Levitt

**Affiliations:** 1 Division of Diabetic Medicine and Endocrinology, Department of Medicine, University of Cape Town, Cape Town, South Africa; 2 Division of Clinical Pharmacology, Department of Medicine, University of Cape Town, Cape Town, South Africa; 3 UCT/MRC Research Unit for Exercise Science and Sports Medicine, Department of Human Biology, University of Cape Town, Cape Town, South Africa; 4 MRC/Wits Developmental Pathways for Health Research Unit, Department of Paediatrics, Faculty of Health Sciences, University of the Witwatersrand, Johannesburg, South Africa; Van Andel Institute, UNITED STATES

## Abstract

**Purpose:**

We determined the prevalence and correlates of low bone mineral density (BMD) in HIV-infected South Africans as there is a paucity of such data from Africa.

**Methods:**

BMD and serum 25-hydroxyvitamin D were measured in HIV-positive participants on antiretroviral therapy (ART) and in those not yet on ART (ART-naïve).

**Results:**

We enrolled 444 participants [median age 35(IQR: 30, 40) years; 77% women]. BMD was low (z score <-2SD) in 17% and 5% of participants at the lumbar spine and total hip, respectively. Total hip [0.909 (SD 0.123) vs 0.956 (SD 0.124) g/cm^2^, p = 0.0001] and neck of femur BMD [0.796 (SD 0.130) vs 0.844 (SD 0.120) g/cm^2^, p = 0.0001] were lower in the ART, compared to the ART-naïve group. Vitamin D deficiency was present in 15% of participants and was associated with efavirenz use [adjusted OR 2.04 (95% CI 1.01 to 4.13)]. In a multivariate linear regression, exposure to efavirenz or lopinavir-based ART was associated with lower total hip BMD, whereas higher weight, being male and higher vitamin D concentration were associated with higher total hip BMD (adjusted R^2^ = 0.28). Age, weight, sex, and the use of efavirenz-based ART were independently associated with lumbar spine BMD (adjusted R^2^ = 0.13).

**Conclusions:**

Vitamin D status, use of efavirenz or lopinavir/ritonavir, weight, age and sex are significantly associated with lower BMD in this young cohort of HIV-infected South Africans.

## Introduction

The prevalence of low bone mineral density (BMD) and the risk of associated fractures are higher in HIV-infected patients compared to the general population[1]. The aetiology of low BMD in HIV infection is multifactorial and includes traditional risk factors (hypogonadism, smoking, ethanol use, sedentary lifestyle, vitamin D deficiency) and HIV-associated factors [antiretroviral therapy (ART) used, initiation of ART, CD4 count at initiation of ART,][2, 3]. Although specific ARTs have been shown to decrease BMD by different mechanisms, all ART regimens studied have been shown to decrease BMD by varying degrees[4].

The World Health Organization’s (WHO) recommended first-line ART regimen includes efavirenz, which reduces vitamin D concentrations by inducing its metabolism[5, 6]. Cumulative use of stavudine which, until recently, was part of first-line ART in resource-limited settings, has been shown to reduce BMD [5, 7]. Tenofovir, which has replaced stavudine in first-line ART WHO recommendations, also reduces BMD[8]. Furthermore, the WHO recommended second-line ART regimen includes a ritonavir-boosted protease inhibitor, usually lopinavir, which has also been associated with a low BMD and an increased risk of fractures [1, 8]. This risk is also likely to increase as the population ages.

However, most of the data on factors associated with low BMD in HIV infection are derived from studies conducted in high-income countries and may not be applicable to middle- or low-income countries where social, nutritional and HIV-associated factors are likely to be different. Furthermore, the majority of people living with HIV in sub-Saharan Africa are young pre-menopausal women[9], who generally have a low risk of developing osteoporosis and who are under-represented in published studies. Vitamin D deficiency is a well-established secondary cause of osteoporosis in HIV-infected and HIV-uninfected people ([10]), and a recent South African study has shown that vitamin D deficiency is linked to poverty and is more common in the HIV-infected South African population[11].

The aims of our study were to determine the prevalence and risk factors for low BMD in a relatively young group of HIV-infected South African men and women.

## Methods

### Participants

HIV-infected patients managed at a community healthcare clinic in Crossroads, Cape Town were conveniently sampled as described previously [12]. The sample included both ART-naïve participants and participants on ART. The study was conducted between 2007 and 2011. The standard first-line regimen in South African National Department of Health protocols at the time comprised stavudine, lamivudine and either efavirenz or nevirapine, with a second-line ART regimen of zidovudine, didanosine and ritonavir-boosted lopinavir (lopinavir/ritonavir), with drug substitutions allowed for intolerance. Patients with the following were excluded: a history of diabetes mellitus or impaired glucose tolerance, ART for < 6 months, an active acute opportunistic infection, severe diarrhoea (>6 stools/day), tuberculosis within 1 month of commencing treatment, glucocorticoid therapy within the past 6 months, pregnant, or known to be in renal failure. The study was approved by the Research Ethics Committee of the Faculty of Health Sciences of the University of Cape Town. Prior to participating in the study, procedures and risks were explained to the subjects, who gave written informed consent to participate in the study.

### Testing procedures

Trained field workers administered a questionnaire to the participants to obtain data on socio-demographic details, current smoking, alcohol ever, physical activity[13], contraception and current medication. Subjects’ clinical records were reviewed and information was extracted on ART regimen, time on ART and current CD4 count. Weight and height were measured and a fasting blood sample drawn. The serum was stored at -80°C for the subsequent analysis of 25-hydroxyvitamin D (25-OH vitamin D) concentrations.

### Biochemical Analyses

25-OH vitamin D levels were determined using the Elecsys Vitamin D electrochemiluminescence assay. Participants were categorised as vitamin D deficient if the serum 25-OH vitamin D concentration was less than 20 ng/mL. Season of sampling for vitamin D concentrations was categorised as winter if the sample was taken between April and September, and summer if the sample was taken between October and March.

### Bone mineral density (BMD)

Whole body (WB), femoral neck (FN), total hip (PF) and lumbar spine (LS) BMD, as well as whole body fat mass (WBFM) and lean mass (fat free soft tissue mass, WBLM) were measured using dual-energy X-ray absorptiometry (DXA) (Hologic Discovery-W, software version 12.7, Hologic Bedford Inc., Bedford, MA, USA).

Due to the wide age range and the inclusion of men and women in the sample, a Z-score (defined as an individuals’ BMD in comparison to age-matched normals) of less than -2 SD was selected to classify individuals as having a low BMD. Participants were categorised as having low BMD at the hip if the total hip or femoral neck Z-score was less than -2 SD at either hip. Participants were categorised as having low BMD at the lumbar spine if the z score for total L1-L4 was less than -2.

### Statistical analysis

All analyses were conducted using STATA version 11 (STATA Corporation, College Station, TX, USA). Continuous variables were described using mean and standard deviation (SD) if normally distributed and median value with inter-quartile range (IQR) if not normally distributed. For linear regression analyses, associations with left total hip BMD and LS BMD were explored. For linear regression analyses of associations with BMD, age, weight and sex were included in the models *a priori*. Models were built using a forward fitting approach, testing all variables with p<0.2, and starting with adding those with the biggest R^2^ on univariate analysis. Variables were left in the model if they improved the model fit and/or were calculated to have a coefficient p<0.1.

For logistic regression of associations with low BMD, age, weight and sex were included in the model *a priori*. Multivariate models were built using a forward fitting approach, first adding those most strongly associated on univariate analysis. All variables with p<0.2 on univariate analysis were tested for inclusion in the model. The decision to keep a variable in the model was based on a likelihood ratio test, using p<0.1 as the cut off for inclusion.

Participants were categorised into an antiretroviral exposure group (efavirenz-based ART, nevirapine based ART or lopinavir-based ART) based on their current antiretroviral therapy regimen. Participants taking an NRTI-sparing regimen comprising lopinavir and either nevirapine or efavirenz were excluded from the analysis of associations between ART exposure group and BMD as well as Vitamin D status.

## Results

There were 444 participants with BMD data at the lumbar spine, total hip, or both sites, and 340/444 (77%) were women. Participant characteristics and antiretroviral exposure are summarised in Table 1. ART regimen data was missing for 10 participants.

**Table 1:** Participant characteristics (n = 444).

Characteristic	All participants	Men (n = 104)	Women (n = 340)
Age (years)	35 (30, 40)	37 (33, 45)	33 (29, 38)
Height (metres)*	1.60 (1.56, 1.65)	1.69 (1.64, 1.73)	1.59 (1.54, 1.63)
BMI (kg/m^2^)*	25.3 (21.8, 30.1)	22.3 (20.6, 24.3)	26.4 (23.0, 31.9)
Alcohol consumption ever n (%)*	203 (48)	84 (86)	119 (36)
Current smoker n (%)*	78 (18)	55 (56)	23 (7)
Lifetime walking (mins/week)	238 (163, 345)	216 (150, 307)	241 (168, 349)
Current CD4 count *	336 (205, 500)	283 (176, 479)	350 (212, 503)
Injectable contraception n (%)*	NA	NA	132 (49)
ART naïve n (%)*	201 (45)	47 (45)	154 (45)
Efavirenz-based ART n (%)	72 (16)	22 (21)	50(15)
Nevirapine-based ART n (%)	74 (17)	16(15)	58 (17)
Lopinavir-based ART n (%)	80 (18)	17 (15)	64 (19)

Continuous data presented as median (IQR).

*differing sample size due to missing data

### Bone mineral density results

BMD results at the lumbar spine and total hip are presented in Table 2.

**Table 2:** Bone mineral density parameters by sex and ART exposure.

	All participants	Men	Women	p value	On ART	Not on ART	p value
Lumbar spine	0.930 (0.120)	0.943 (0.123)	0.928 (0.119)	0.408	0.925 (0.125)	0.937 (0.112)	0.287
Femoral neck	0.817 (0.128)	0.820 (0.136)	0.816 (0.125)	0.798	0.795 (0.130)	0.844 (0.120)	<0.0001
Total hip	0.930 (0.125	0.973 (0.128)	0.917 (0.121)	0.0001	0.908 (0.122)	0.956 (0.124)	<0.0001

Total hip and femoral neck BMD were significantly lower in participants on ART, compared to ART-naïve participants but lumbar spine BMD did not differ significantly between these 2 groups (Table 2). Of 435 with a Z-score result for the lumbar spine, 73/435 (17%) had low BMD. BMD was low at the total hip in 22/440 (5%) of the participants.

### Vitamin D results

We measured Vitamin D concentrations in 430 participants with stored plasma available for analysis. The median 25-OH vitamin D concentration was 33 ng/mL (IQR 24 to 44), and 65/430 (15%) participants were classified as vitamin D deficient. There was a seasonal difference in 25-OH vitamin D concentration, with 49/259 (19%) of those sampled in winter and 16/169 (9%) of those sampled in summer being classified as vitamin D deficient (Chi squared p = 0.008).

The proportion of participants with vitamin D deficiency differed significantly between ART exposure groups: 17/70 (24%) of those on efavirenz-based ART, 12/71(17%) of those on nevirapine-based ART, and 7/78 (9%) of those on lopinavir-based ART were vitamin D deficient (Chi squared p = 0.043). In a multivariate logistic regression model (not shown), after adjustment for age, sex and season of sampling, efavirenz use remained significantly associated with vitamin D deficiency, OR 2.04 (95% CI: 1.01 to 4.13).

### Associations with total hip BMD

Univariate analyses and the multivariate linear regression model of associations with total hip are presented in Table 3 (n = 412). Exposure to efavirenz or lopinavir-based ART, but not nevirapine-based ART, was independently associated with lower total hip BMD, compared to ART naïve participants. Higher weight, being male and higher vitamin D concentration were independently associated with higher total hip BMD. We found no association between total hip BMD and alcohol consumption, smoking, physical activity, current CD4 count, or using the injectable contraceptive (in women participants).

**Table 3:** Multivariate linear regression model of associations with total hip BMD (g/cm^2^) n = 412*.

Univariate analysis			Multivariate analysis	
Variable	Beta coefficient (95% CI)	p-value	Beta coefficient (95% CI)	p-value
Age (years)**	-0.002 (-0.016 to 0.013)	0.833	-0.007(-0.021 to 0.005)	0.249
Weight (kg)**	0.028 (0.022 to 0.035)	<0.001	0.033 (0.027 to 0.039)	<0.0001
Male	0.057 (0.029 to 0.085)	<0.001	0.081 (0.055 to 0.106)	<0.0001
Vit D (nmol/L)*	0.008 (0.000 to 0.016)	0.058	0.008 (0.000 to 0.015)	0.037
ART exposure				
ART naive	referent		referent	
efavirenz–based ART	-0.071 (-0.105, -0.037)	<0.0001	-0.075(-0.105 to -0.044)	<0.0001
nevirapine-based ART	-0.025 (-0.059, 0.008)	0.141	-0.021(-0.050 to 0.008)	0.158
lopinavir–based ART	-0.057 (-0.089, -0.024)	0.001	-0.067(-0.095 to -0.038)	<0.0001

Model adjusted R^2^ = 0.28.

*univariate and multivariate analyses calculated on 412 participants with complete data for all variables.

** increments of 10.

When the same analysis was performed restricted to participants taking ART (n = 201), higher baseline CD4 count was independently associated with higher total hip BMD. Associations with ART exposure were similar, but Vitamin D was no longer significantly associated with BMD (model not shown).

### Associations with low total hip BMD

In a multivariate logistic regression model (n = 423), with age, sex and weight included *a priori* in the model, weight and efavirenz or lopinavir exposure were strongly and independently associated with low BMD at the hip (z score < -2SD at either the total hip or femoral neck) (Table 4).

**Table 4:** Multivariate logistic regression model of associations with low bone density (Z-score < -2 SD) at the hip.

Variable	Number (%)	Crude odds ratio (95% CI)	Adjusted odds ratio (95% CI)	Wald test p value
Age**	-	0.96 (0.57 to 1.62)	0.81 (0.43 to 1.50)	0.495
Sex Female Male	325 (76)101 (24)	10.77 (0.24, 2.15)	10.50 (0.13 to 1.94)	0.317
Weight***	-	0.34 (0.20 to 0.57)	0.33 (0.19 to 0.55)	<0.001
Antiretroviral therapy ART naïve Efavirenz-based ART Nevirapine-based ART Lopinavir-based ART		200 (47)72 (17)74 (17)80 (19)	14.17 (1.28 to 13.62)1.07 (0.24 to 5.68)3.23 (0.96 to 10.92)	15.71 (1.56 to 20.95)1.46 (0.26 to 8.05)5.81 (1.52 to 22.11)	0.0090.6670.010

n = 423*.

*crude and adjusted ORs calculated for the 423 participants with complete data for all variables included in the multivariate model.

**included in the model as a continuous variable. The odds is for a 10 year increase.

***included in the model as a continuous variable. The odds is for a 10 kg increase.

### Associations with lumbar spine BMD

Univariate analyses and the multivariate linear regression model (n = 423) of associations with lumbar spine BMD are presented in Table 5. Weight and being a male were positively associated with lumbar spine BMD, and age, and use of efavirenz-based ART were negatively associated with lumbar spine BMD. Nevirapine and lopinavir-based ART use were not associated with lumbar spine BMD. We found no association between lumbar spine BMD and alcohol consumption, smoking, physical activity, current CD4 count or Vitamin D concentration.

**Table 5:** Multivariate linear regression model of associations with lumbar spine BMD (g/cm^2^), n = 423*.

Univariate analysis			Multivariate analysis	
Variable	Beta coefficient (95% CI)	p-value	Beta coefficient (95% CI)	p-value
Age (years)**	-0.018 (-0.031 to -0.005)	0.005	-0.023 (-0.036 to -0.010)	<0.001
Weight (kg)**	0.021 (0.014 to 0.027)	<0.001	0.023 (0.017 to 0.029)	<0.001
Male	0.013 (-0.014 to 0.040)	0.339	0.039 (0.013 to 0.065)	0.003
ART exposure				
ART naive	referent		referent	
efavirenz–based ART	-0.035 (-0.067 to -0.003)	0.034	-0.032 (-0.062 to -0.001)	0.040
nevirapine-based ART	0.006 (-0.026 to 0.038)	0.722	0.007 (-0.023 to 0.037)	0.655
lopinavir–based ART	-0.017 (-0.048 to 0.014)	0.283	-0.021 (-0.050 to 0.009)	0.169

Model adjusted R^2^ = 0.13

*univariate and multivariate analyses calculated on 423 participants with complete data for all variables. 19 participants were missing data for 1 or more variables included in the multivariate model.

**increments of 10.

### Associations with low lumbar spine BMD

In a multivariate logistic regression model (n = 418) after adjustment for sex, the odds of low lumbar spine BMD was decreased with a greater body weight, with an adjusted OR of 0.57 (95% CI 0.45 to 0.71) for every additional 10 kg of weight (Wald test p<0.001).

## Discussion

In this cross-sectional sample of South African HIV-infected patients, we found that a low BMD at the lumbar spine in 17% of participants and low BMD at the hip in 5% of participants. Vitamin D deficiency was found in 15% of participants and the prevalence of vitamin D deficiency was higher in those participants who had been exposed to efavirenz. In addition, the use of efavirenz or lopinavir was associated with a greater odds of having a low BMD at the total hip, and both were independently associated after adjustment for age, weight, sex and vitamin D concentration. The use of efavirenz was also inversely associated with BMD at the LS, after adjustment for age, sex and weight.

Although there are some conflicting reports on whether HIV-infected men have a lower BMD than HIV-negative men[14, 15], there is consistent international data showing that HIV-infected women, both pre-menopausal and post-menopausal, have a lower BMD than HIV-negative women, and that this is accompanied by microarchitectural changes at the hip and lumbar spine[16, 17]. There is limited data on the prevalence and predictors of low BMD in HIV-infected individuals from developing countries. A recent report on ART-naïve urban black South African women with HIV infection shows that they did not have a lower BMD or lower 25 hydroxyvitamin D concentrations when compared with HIV-negative controls[18]. In contrast, Senegalese HIV-infected men and women had reduced BMD, assessed using quantitative ultrasound at the calcaneus, when compared with age- and sex-matched controls from the general population[19].

Due to a higher trabecular content, a low BMD at the lumbar spine is more often due to a metabolic cause rather than advancing age or insufficient mechanical loading, which is more often the cause of a low BMD at the hip, an area of primarily cortical bone. Since the participants in our study were relatively young a 5% prevalence of low BMD at the hip was not unexpected. However, our study cannot adequately explain the higher prevalence we observed of low BMD at the lumbar spine. A low BMD in the setting of HIV infection has been commonly ascribed to traditional risk factors (such as low BMI, smoking and older age) for osteoporosis and factors specific to HIV-infection such as the ART regimen used, initiation of ART and CD4 count at initiation of ART[20].

We found an association between efavirenz and lopinavir use and lower hip BMD but we did not find an association between BMD and nevirapine exposure. This may be due to lack of power, increased magnitude of the effect of efavirenz, or residual confounding by an unmeasured variable. A cross-sectional study reported that protease inhibitors, but not non-nucleoside reverse transcriptase inhibitors, were associated with low BMD[21]. However, a sub-study of a randomised controlled trial found similar decreases in total BMD with efavirenz- and lopinavir-based ART[22], in keeping with our findings. It has been hypothesised that BMD loss that occurs after ART initiation is an immune reconstitution phenomenon, and is therefore independent of specific ART regimens[23]. In addition, efavirenz has been shown to be independently associated with Vitamin D deficiency[5, 24, 25], a finding confirmed in our study, and Vitamin D deficiency is known to be common in black South Africans with HIV infection[11]. It has been hypothesized that efavirenz causes the induction of cytochrome P450 isoenzyme CYP3A4 (which converts vitamin D to 25-hydroxy-Vitamin D, thereby reducing the amount of Vitamin D substrate available), as well as CYP24, which converts 25-hydroxy-Vitamin D to an inactive metabolite[5, 24]. In our study efavirenz use was associated with a low BMD at the hip and lumbar spine independent of Vitamin D status in the multivariate linear regression models, therefore the effect of efavirenz on BMD may be mediated by both an effect on Vitamin D and an additional, independent mechanism.

Our study has several limitations. The data are cross-sectional, so we could not explore changes in BMD over time. The cross sectional design allows us to characterise risk factors, but not to determine causality. Since we used convenience sampling we cannot rule out sampling bias. We did not have data on previous history of fractures, and could not calculate fracture risk for participants.

The clinical implications of our study findings require further exploration. A recent systematic review confirmed that HIV infection was associated with a modest increase in fracture risk, and suggested that race may modify fracture risk, with non-black HIV-infected people being at higher risk than black HIV-infected people[26]. Studies assessing the influence of HIV infection and ART exposure on the risk of fractures in an African population are required.

In conclusion, our study shows that Vitamin D status, use of efavirenz or lopinavir/ritonavir, weight, age and sex are significantly associated with lower BMD in this young cohort of HIV-infected South Africans. Further studies exploring the effect of the combination of efavirenz and tenofovir on BMD should be done, especially as the current World Health Organisation guidelines recommend the combination of tenofovir, emtricitabine/lamivudine and efavirenz as the standard first line for patients initiating ART.

## Supporting Information

S1 Dataset Data used to generate results in this manuscript. (XLSX)
